# Determinants of time to full enteral nutrition and its association with short-term outcomes in very and extremely low birth weight infants: a multicenter study in China

**DOI:** 10.1186/s12887-026-07102-z

**Published:** 2026-07-08

**Authors:** Min Yu, Xingxing Wu, Hong Li, Jian Yu

**Affiliations:** Department of Neonatology, Children’s Hospital of Soochow University, Suzhou, China

**Keywords:** Extremely low birth weight, Full enteral feeding, Neonatal outcomes, Short-term complications, Very low birth weight

## Abstract

**Background:**

Achieving full enteral nutrition (FEN) is a critical milestone for very low birth weight (VLBW) and extremely low birth weight (ELBW) infants. However, determinants of FEN timing and its clinical implications remain insufficiently characterized in China. To identify factors associated with time to FEN and evaluate its relationship with short-term outcomes in VLBW and ELBW infants.

**Methods:**

This multicenter retrospective study included 773 preterm infants admitted between January 2019 and June 2023. Infants were stratified into VLBWI (*n* = 659) and ELBWI (*n* = 114). Clinical characteristics, feeding progression, biochemical markers, and clinical outcomes were analyzed. Multivariable generalized linear models were used to identify factors independently associated with time to FEN.

**Results:**

In VLBWI, lower gestational age, lower birth weight, and treatment-requiring patent ductus arteriosus (PDA) were independently associated with delayed FEN (*P* < 0.05). Delayed FEN was associated with increased risks of necrotizing enterocolitis, bronchopulmonary dysplasia, late-onset sepsis, and severe retinopathy of prematurity, as well as higher levels of cholestasis-related biomarkers. No statistically significant associations were identified in ELBWI. Although infants with prolonged FEN demonstrated higher discharge anthropometric measurements, these findings should be interpreted cautiously because of differences in hospitalization duration.

**Conclusions:**

In VLBWI, delayed achievement of full enteral nutrition was associated with developmental immaturity, hemodynamic instability, and increased short-term morbidity. Time to FEN may serve as a clinically meaningful marker of overall physiological instability rather than an isolated causal determinant of adverse outcomes. Prospective multicenter studies with standardized feeding protocols are warranted to further clarify the relationship between feeding progression and neonatal outcomes.

## Introduction

Very low birth weight infants (VLBWI) are highly vulnerable to postnatal nutritional deficits after birth due to limited nutrient reserves, gastrointestinal immaturity, and common delays in the initiation and advancement of enteral feeding [1, 2]. As a result, extrauterine growth restriction (EUGR) remains highly prevalent in this population and is strongly associated with adverse neurodevelopmental outcomes and a heightened risk of metabolic disorders later in life [3–5]. Accordingly, early optimization of enteral nutrition and timely progression from parenteral nutrition to full enteral feeding (FEN) are recognized as essential components of contemporary neonatal care [6].

However, advancing enteral feeding in VLBWI presents inherent clinical challenges. Slow feed progression prolongs reliance on parenteral nutrition and increases the risk of infection, cholestasis [7], and whereas overly rapid advancement may trigger metabolic complications [8–10]. Although current guidelines provide broad recommendations for the initiation and advancement of enteral feeds, substantial variability persists in real-world practice, and many VLBWI reach FEN substantially later than guideline-based expectations [11]. Furthermore, while previous studies have examined different feeding strategies, the influence of maternal perinatal factors and early neonatal conditions on the timing of FEN remains insufficiently understood.

In China, enteral feeding practices vary considerably among neonatal intensive care units, including differences in human milk availability, feeding advancement strategies, parenteral nutrition practices, and management of hemodynamically significant patent ductus arteriosus (hsPDA). Although several domestic studies have evaluated feeding tolerance and nutritional management in preterm infants, few have comprehensively examined factors associated with time to FEN while simultaneously evaluating short-term morbidities, biochemical indicators of parenteral nutrition–associated hepatic stress, and early growth outcomes. Therefore, additional real-world multicenter data are needed to better characterize feeding progression in Chinese neonatal settings.

In this multicenter retrospective cohort study, we analyzed VLBWI and ELBWI admitted to two tertiary neonatal centers in Suzhou. The objectives of the study were to: (1) identify perinatal and neonatal factors associated with time to full enteral feeding; and (2) evaluate the associations between delayed FEN and short-term clinical outcomes, biochemical markers, and early postnatal growth. By examining feeding progression across different TFF intervals in both VLBWI and ELBWI populations, this study aimed to provide clinically relevant observational evidence that may help inform individualized nutritional management strategies for high-risk preterm infants.

## Materials and methods

### Study design and participants

This multicenter retrospective cohort study included preterm infants admitted to the neonatal intensive care units (NICUs) of Suzhou Municipal Hospital and the Children’s Hospital of Soochow University from January 2019 to June 2023.

Inclusion criteria were birth weight < 1500 g and admission within 24 h after birth. Exclusion criteria included death or discharge before achieving FEN, major congenital anomalies, gastrointestinal malformations requiring surgery, and confirmed metabolic disorders.

Ethical approval was obtained from the Ethics Committee of the Children’s Hospital of Soochow University (Approval No. 2023CS126). Informed consent was waived due to the retrospective nature of the study and the use of de-identified data.

### Grouping strategy

Infants were categorized according to birthweight as follows: extremely low birth weight infants (ELBWI) < 1000 g and very low birth weight infants (VLBWI) 1000–1499 g.

Time to full enteral feeding (TFF) was defined as the duration from birth to the first sustained enteral intake of ≥ 150 mL/kg/day for at least 24 h. The participating NICUs followed broadly similar principles for enteral feeding advancement based on institutional protocols and clinical assessment. However, detailed feeding practices, including feeding advancement rates, timing of fortification, feeding interruption criteria, and use of human milk or donor milk, were not fully standardized across centers. Feeding decisions were ultimately individualized according to the infant’s clinical condition and treating physician judgment.

Based on the Canadian VLBW Feeding Guidelines, infants were stratified into the following TFF groups: ELBWI: >14–21 days, > 21–28 days, and > 28 days; VLBWI: >7–14 days, > 14–21 days, > 21–28 days, and > 28 days.

### Data collection

Data were extracted from electronic medical records and included the following variables:

- Baseline characteristics: Infant: sex, gestational age (GA), birth weight, head circumference at birth, mode of delivery, small-for-gestational-age (SGA) status, and 5-min Apgar score. Maternal: hypertensive disorders of pregnancy, gestational diabetes mellitus, and antenatal corticosteroid exposure.
- Primary outcomes: time to FEN; major morbidities including NEC, bronchopulmonary dysplasia (BPD), late-onset sepsis (LOS), and severe retinopathy of prematurity (sROP requiring treatment); and peak biochemical markers within the first 4 weeks, alkaline phosphatase (ALP), alanine aminotransferase (ALT), and conjugated bilirubin). Secondary outcomes: anthropometric measurements (weight and head circumference) at day 28, corrected gestational age (CGA) 36 weeks, and discharge.

### Definitions

NEC was defined as stage ≥ IIA [12]. BPD was defined using the optimal definition of Jensen et al. [13]. LOS was defined as blood or cerebral spinal fluid culture-confirmed sepsis after 3 days of age [14]. hemodynamically significant persistent ductus arteriosus (hsPDA) was defined based on echocardiographic findings and clinical hemodynamic significance [15]. sROP was defined as stage ≥ 3 including receipt of intervention and/or treatment [16]. Disease diagnosis was based on the International Classification of Diseases, 10th Revision (ICD-10).

### Statistical analysis

Statistical analyses were performed using SPSS version 27.0. Continuous variables were expressed as mean ± standard deviation (SD) and compared using the independent-samples t-test when normally distributed or the Wilcoxon rank-sum test when non-normally distributed. Categorical variables were presented as frequencies and percentages and compared using the χ² test or Fisher’s exact test. Generalized linear models (GLMs) were applied to identify clinical factors independently associated with time to full enteral feeding. Variables with clinical relevance or *P* < 0.10 in univariable analyses were entered into multivariable models. A two-tailed *P* value < 0.05 was considered statistically significant.

**Fig. 1:**
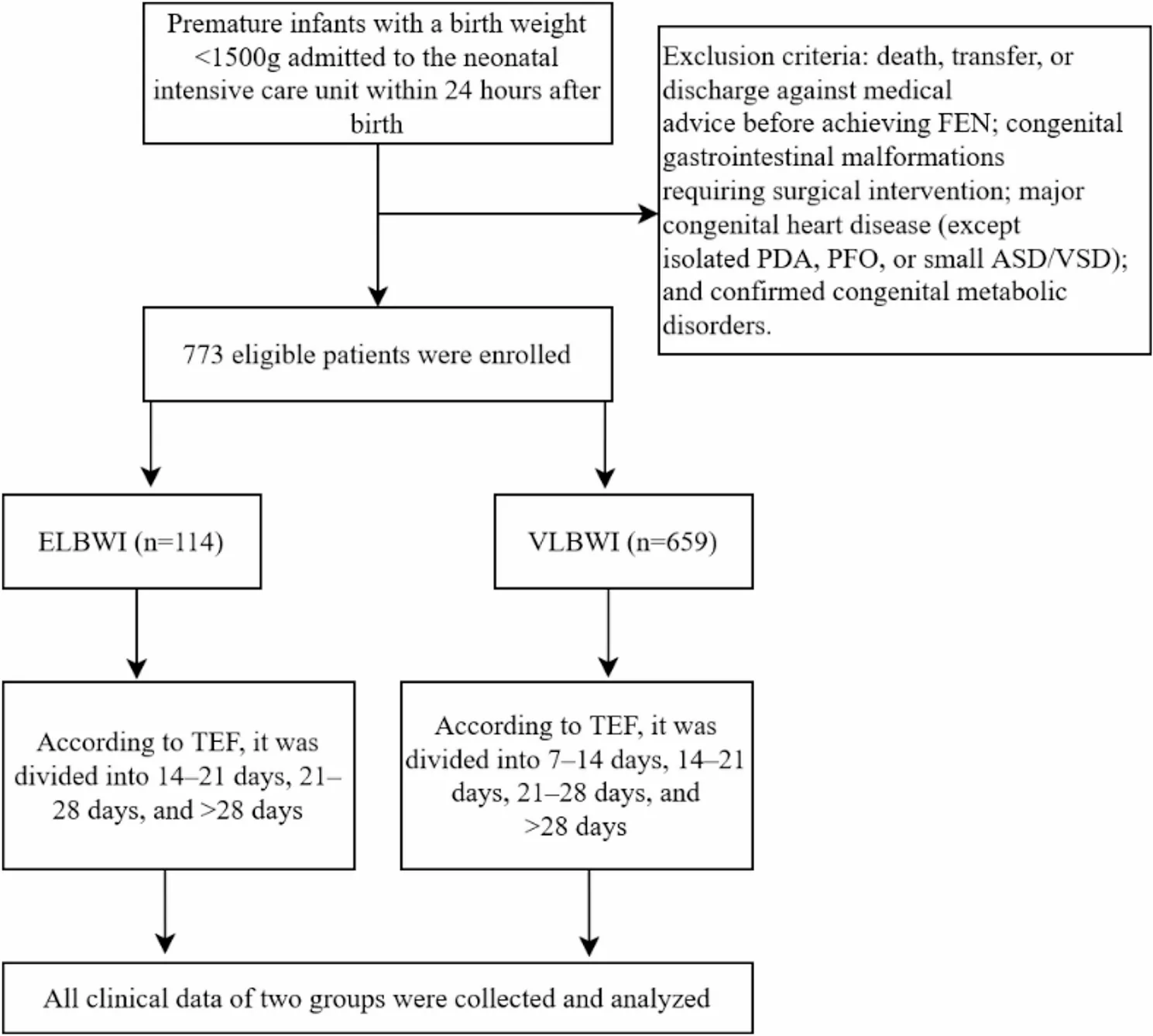
Study flowchart illustrating inclusion and exclusion of infants with birthweight<1500 g across two participating NICUs

Because major neonatal morbidities may both influence and be influenced by feeding progression, the analyses were designed to evaluate clinical associations rather than establish causal relationships (Fig. 1).

## Results

### Baseline characteristics and predictors of TFF

A total of 773 infants were included in the analysis, comprising 114 ELBWI and 659 VLBWI. Compared with VLBWI, ELBWI had significantly lower gestational age, higher rates of SGA, lower 5-min Apgar scores, longer TFF, and longer hospitalization (all *P* < 0.05). The mean TFF was 42.31 ± 18.09 days in ELBWI and 28.78 ± 14.86 days in VLBWI. No significant differences were observed in sex distribution or other baseline characteristics (Table 1).

**Table 1:** Comparison of clinical characteristics between infants with birth weight < 1000 g and 1000–1499 g

Variable	< 1000 g (*n* = 114)	1000–1499 g (*n* = 659)	t/χ²	*P* value
Gestational age (weeks)	28.12 ± 2.16	30.08 ± 1.82	−8.671	< 0.001
Male sex, n (%)	60 (52.6)	345 (52.4)	0.003	0.956
Cesarean section, n (%)	49 (43.0)	303 (46.0)	0.352	0.553
Multiple birth, n (%)	35 (30.7)	154 (23.4)	2.829	0.093
SGA, n (%)	52 (45.6)	152 (23.1)	25.437	< 0.001
5-min Apgar score	8.30 ± 1.41	8.89 ± 1.19	−5.446	< 0.001
Maternal hypertensive disorders, n (%)	36 (31.6)	192 (29.1)	0.279	0.597
PDA requiring treatment, n (%)	23 (20.2)	115 (17.5)	0.641	0.423
Time to full enteral feeding (days)	42.3 ± 18.09	28.7 ± 14.86	−7.397	< 0.001
Length of hospital stay (days)	75.81 ± 21.19	50.05 ± 16.01	−11.735	< 0.001

Continuous variables are presented as mean ± standard deviation and compared using the independent samples t-test. Categorical variables are expressed as number (percentage) and compared using the chi-square test

A two-tailed *P* < 0.05 was considered statistically significant

*Abbreviations*: *SGA* Small for gestational age, *PDA* Patent ductus arteriosus

In ELBWI, no statistically significant associations were identified between TFF and any of the examined variables in ELBWI, including gestational age, birthweight, sex, mode of delivery, maternal hypertension, multiple gestation, antenatal corticosteroid exposure, and Apgar score (Table 2).

**Table 2:** Multivariable generalized linear model analysis of factors associated with time to full enteral feeding (TFF) in extremely low birth weight infants

Variable	Coefficient (β)	Standard Error	t value	*P* value	95%CI Lower	95%CI Upper
Intercept	43.8790	56.212	0.781	0.435	−66.294	154.052
Gestational age	−0.6353	1.743	−0.365	0.715	−4.051	2.780
Birth weight	−0.0142	0.028	−0.517	0.605	−0.068	0.040
Male sex	−0.0847	4.377	−0.019	0.985	−8.664	8.494
SGA	0.3706	7.529	0.049	0.961	−14.385	15.126
NRDS	−0.7122	5.946	−0.120	0.905	−12.366	10.941
Multiple birth	2.6566	4.913	0.541	0.589	−6.973	12.286
Cesarean section	1.5383	4.606	0.334	0.738	−7.489	10.566
Antenatal steroids (> 1 dose)	1.4621	4.522	0.323	0.746	−7.401	10.325
PDA requiring treatment	4.9853	5.311	0.939	0.348	−5.425	15.395
5-min Apgar score	0.1968	1.507	0.131	0.896	−2.757	3.151

A generalized linear model was used to identify independent factors associated with TFF. Results are presented as regression coefficients (β) with 95% confidence intervals (CI)

A two-tailed *P* < 0.05 was considered statistically significant

*Abbreviations*: *SGA* Small for gestational age, *NRDS* Neonatal respiratory distress syndrome, *PDA* Patent ductus arteriosus, *TFF* Time to full enteral feeding

In contrast, among VLBWI, lower gestational age (β = − 2.286, *P* < 0.001), lower birthweight (β = − 0.018, *P* = 0.001), and treatment-requiring hsPDA (β = 3.86, *P* = 0.008) were significantly associated with longer TFF. Other variables, including sex, delivery mode, and maternal complications, were not associated with TFF (Table 3).

**Table 3:** Multivariable linear regression analysis of factors associated with time to full enteral feeding (TFF) in very low birth weight infants

Variable	β	SE	t value	*P* value	95% CI
Intercept	126.536	16.469	7.683	< 0.001	94.257 to 158.814
Gestational age (weeks)	−2.286	0.555	−4.118	< 0.001	−3.375 to − 1.198
Birth weight (g)	−0.018	0.005	−3.478	0.001	−0.028 to − 0.008
Male sex	0.547	1.122	0.487	0.626	−1.653 to 2.746
SGA	−0.927	1.997	−0.464	0.643	−4.840 to 2.987
NRDS	−1.051	1.257	−0.836	0.403	−3.515 to 1.412
Multiple birth	−1.806	1.295	−1.395	0.163	−4.344 to 0.731
Cesarean section	2.230	1.192	1.871	0.061	−0.106 to 4.566
Antenatal steroids (> 1 dose)	−0.705	1.132	−0.623	0.533	−2.925 to 1.514
PDA requiring treatment	3.860	1.457	2.650	0.008	1.005 to 6.715
5-min Apgar score	0.487	0.491	0.992	0.321	−0.475 to 1.449

Multiple linear regression analysis was performed to identify independent factors associated with TFF in very low birth weight infants. Regression coefficients (β) with 95% confidence intervals (CI) are presented

A two-tailed *P* value < 0.05 was considered statistically significant

*Abbreviations*: *SGA* Small for gestational age, *NRDS* Neonatal respiratory distress syndrome, *PDA* Patent ductus arteriosus, *TFF* Time to full enteral feeding

### Short-term outcomes across TFF groups

Among ELBWI, no statistically significant differences were observed across the three TFF groups in the incidence of NEC, BPD, LONS, sROP, or in peak biochemical markers (all *P* > 0.05) (Tables 4 and 5).

**Table 4:** Comparison of short-term complications according to time to full enteral feeding (TFF) in extremely low birth weight infants (< 1000 g)

TFF (days)	*n*	NEC, *n* (%)	BPD, *n* (%)	LONS, *n* (%)	ROP requiring treatment, *n* (%)
14 < d ≤ 21	9	0 (0.0)	8 (88.9)	1 (11.1)	3 (33.3)
21 < d ≤ 28	22	1 (4.5)	20 (90.9)	3 (13.6)	6 (27.3)
d > 28	83	10 (12.0)	77 (92.8)	16 (19.3)	34 (41.0)
χ²	—	1.207	0.819	0.413	1.468
*P* value	—	0.470	0.591	0.917	0.480

Categorical variables were compared using the chi-square test or Fisher’s exact test, as appropriate.

A two-tailed *P* value < 0.05 was considered statistically significant

*Abbreviations*: *NEC* Necrotizing enterocolitis, *BPD* Bronchopulmonary dysplasia, *LONS* Late-onset neonatal sepsis, *ROP* Retinopathy of prematurity, *TFF* Time to full enteral feeding

**Table 5:** Comparison of short-term complications according to time to full enteral feeding (TFF) in very low birth weight infants (1000–1499 g)

TFF (days)	*n*	NEC, *n* (%)	BPD, *n* (%)	LONS, *n* (%)	ROP requiring treatment, *n* (%)
7 < d ≤ 14	106	0 (0.0)^a^	78 (73.6)^a^	4 (3.8)^a^	4 (3.8)^c^
14 < d ≤ 21	151	4 (2.6)^a^	120 (79.5)^a^ ^b^	12 (7.9)^a^	13 (8.6)^b^ ^c^
21 < d ≤ 28	132	2 (1.5)^a^	111 (84.1)^a^ ^b^	13 (9.8)^a^	19 (14.4)^b^
d > 28	270	26 (9.6)	232 (85.9)^b^	57 (21.1)	40 (14.8)^b^
χ²	—	23.508	8.983	28.159	11.430
*P* value	—	< 0.001	0.030	< 0.001	0.010

Categorical variables were compared using the chi-square test. Post hoc pairwise comparisons were performed with Bonferroni correction

A two-tailed* P* value < 0.05 was considered statistically significant

*Abbreviations*: *NEC* Necrotizing enterocolitis, *BPD* Bronchopulmonary dysplasia, *LONS* Late-onset neonatal sepsis, *ROP* Retinopathy of prematurity, *TFF* Time to full enteral feeding

^a^ *P* < 0.05 vs. d > 28 group

^b^ *P* < 0.05 vs. 7 < d ≤ 14 group

^c^ *P* < 0.05 vs. both 21 < d ≤ 28 and d > 28 groups

Among VLBWI, infants with TFF > 28 days had significantly higher rates of NEC (9.6%), BPD (85.9%), LONS (21.1%), and sROP (14.8%) compared with those who achieved FEN earlier (all *P* < 0.05) (Table 6). These infants also exhibited significantly higher peak levels of ALP, ALT, and conjugated bilirubin (all *P* < 0.05) (Table 7).

**Table 6:** Comparison of biochemical parameters according to time to full enteral feeding (TFF) in extremely low birth weight infants (< 1000 g)

TFF (days)	ALP (U/L)	ALT (U/L)	AST (U/L)	TSB (µmol/L)	CB (µmol/L)	UCB (µmol/L)	GGT (U/L)
14 < d ≤ 21	375.32 ± 140.21	11.45 ± 9.12	36.78 ± 22.34	106.78 ± 50.23	18.12 ± 10.45	88.34 ± 48.67	182.45 ± 108.34
21 < d ≤ 28	382.67 ± 152.78	12.67 ± 14.89	38.91 ± 27.56	104.56 ± 51.34	19.45 ± 13.78	87.23 ± 49.12	170.23 ± 110.45
d > 28	391.45 ± 170.56	14.23 ± 22.34	41.23 ± 35.67	109.89 ± 55.67	21.34 ± 18.45	89.67 ± 52.34	161.78 ± 101.23
F/H value	2.345	1.123	0.987	0.456	1.789	0.234	2.001
P value	0.31	0.571	0.61	0.796	0.409	0.89	0.368

Data are presented as mean ± standard deviation. Comparisons among groups were performed using one-way ANOVA or the Kruskal–Wallis test, as appropriate. Post hoc pairwise comparisons were conducted using Bonferroni correction

A two-tailed *P* < 0.05 was considered statistically significant

*Abbreviations*: *ALP* Alkaline phosphatase, *ALT* Alanine aminotransferase,* AST* Aspartate aminotransferase, *TSB* Total serum bilirubin,* CB* Conjugated bilirubin, *UCB* Unconjugated bilirubin, *GGT* Gamma-glutamyl transferase, *TFF* Time to full enteral feeding

**Table 7:** Comparison of biochemical parameters according to time to full enteral feeding (TFF) in very low birth weight infants (1000–1499 g)

TFF (days)	ALP (U/L)	ALT (U/L)	AST (U/L)	TSB (µmol/L)	CB (µmol/L)	UCB (µmol/L)	GGT (U/L)
7 < d ≤ 14	325.87 ± 72.56^a^	11.37 ± 8.79	33.05 ± 18.46	89.59 ± 47.54	15.14 ± 4.86^b^	75.66 ± 45.83	166.77 ± 102.35
14 < d ≤ 21	361.61 ± 146.20^a^	8.60 ± 6.41	30.23 ± 15.55	94.56 ± 44.95	17.49 ± 5.83	78.05 ± 42.69	182.41 ± 95.49
21 < d ≤ 28	398.43 ± 178.28	11.33 ± 24.61	33.37 ± 30.76	94.87 ± 42.55	19.74 ± 17.11	79.62 ± 40.22	151.18 ± 104.40
d > 28	451.38 ± 200.00	20.16 ± 40.24	45.60 ± 55.08	108.26 ± 55.43	28.12 ± 30.54	87.87 ± 50.29	141.00 ± 82.15
F/H value	14.015	12.655	5.911	3.664	11.988	1.562	7.566
*P* value	0.003	0.005	0.116	0.300	0.007	0.668	0.056

Data are presented as mean ± standard deviation. Comparisons among groups were performed using one-way ANOVA or the Kruskal–Wallis test, as appropriate. Post hoc pairwise comparisons were conducted using Bonferroni correction

A two-tailed *P* < 0.05 was considered statistically significant

*Abbreviation*s: *ALP* Alkaline phosphatase,* ALT* Alanine aminotransferase, *AST* Aspartate aminotransferase, *TSB* Total serum bilirubin, *CB* Conjugated bilirubin, *UCB* Unconjugated bilirubin, *GGT* Gamma-glutamyl transferase,* TFF* Time to full enteral feeding

^a^ *P* < 0.05 vs. d > 28 group

^b^ *P* < 0.05 vs. d > 28 group

#### Anthropometric findings

In ELBWI, only discharge weight differed among the TFF groups, with infants in the > 28 days group exhibiting the highest discharge weight (*P* < 0.05). No statistically significant differences were observed at earlier time points or in head circumference at any time point (Tables 8 and 9).

**Table 8:** Comparison of body weight growth among different TFF groups in extremely low birth weight infants (< 1000 g)

TFF (days)	Birth weight (g)	Weight at day 28 (g)	Weight at 36 weeks PMA (g)	Discharge weight (g)
14 < d ≤ 21	847.78 ± 97.70	1178.75 ± 223.63	1551.25 ± 317.01	2084.44 ± 199.07
21 < d ≤ 28	884.09 ± 105.67	1215.68 ± 228.82	1731.05 ± 305.47	2035.00 ± 359.70^a^
d > 28	866.32 ± 96.85	1231.85 ± 200.13	1770.43 ± 331.17	2330.36 ± 645.22
H/F value	1.965	0.260	1.662	6.317
*P* value	0.374	0.771	0.195	0.042

Data are presented as mean ± standard deviation

Birth weight, weight at birth; Weight at day 28, body weight at postnatal day 28; Weight at 36 weeks PMA, body weight at corrected gestational age of 36 weeks; Discharge weight, body weight at hospital discharge

*P* < 0.05 was considered statistically significant

*TFF* Time to full feeding, *PMA* Postmenstrual age

^a^ *P* < 0.05 compared with the d > 28 group

**Table 9:** Comparison of head circumference growth among different TFF groups in extremely low birth weight infants (< 1000 g)

TFF (days)	Birth HC (cm)	HC at day 28 (cm)	HC at 36 weeks PMA (cm)	Discharge HC (cm)
14 < d ≤ 21	25.42 ± 2.05	26.30 ± 1.50	28.34 ± 1.24	31.22 ± 1.23
21 < d ≤ 28	24.16 ± 1.64	26.43 ± 1.52	29.26 ± 0.82	30.56 ± 1.63
d > 28	24.33 ± 1.97	26.30 ± 1.57	29.28 ± 2.13	31.28 ± 1.71
Statistic (F/H)	2.410	0.036	0.582	1.838
*P* value	0.300	0.965	0.563	0.399

Data are presented as mean ± standard deviation

No significant differences in head circumference growth were observed among the different TFF groups

Birth HC, head circumference at birth; HC at day 28, head circumference at postnatal day 28; HC at 36 weeks PMA, head circumference at corrected gestational age of 36 weeks; Discharge HC, head circumference at hospital discharge

*P* < 0.05 was considered statistically significant

*TFF* time to full feeding, *HC* Head circumference,* PMA* Postmenstrual age

Among VLBWI, longer TFF was associated with lower birthweight and smaller head circumference at birth (*P* < 0.05), and similar differences persisted at day 28. However, by CGA 36 weeks and at discharge, Infants with prolonged TFF had higher absolute weight and head circumference values at discharge; however, these findings should be interpreted cautiously because these infants also had significantly longer hospitalization duration. Differences in discharge anthropometric measures may therefore reflect varying lengths of inpatient nutritional support rather than superior growth trajectories. (Tables 10 and 11).

**Table 10:** Comparison of body weight growth among different TFF groups in very low birth weight infants (1000–1499 g)

TFF (days)	Birth weight (g)	Weight at day 28 (g)	Weight at 36 weeks PMA (g)	Discharge weight (g)
7 < d ≤ 14	1315.63 ± 116.04^a^^b^	1724.79 ± 188.12^c^	1782.16 ± 250.90^de^	2086.57 ± 214.64^f^
14 < d ≤ 21	1281.92 ± 135.88^b^	1695.59 ± 220.48^c^	1869.47 ± 268.68^d^	2121.13 ± 271.36^f^
21 < d ≤ 28	1261.59 ± 142.38	1667.71 ± 235.85	1909.60 ± 325.20^d^	2156.33 ± 376.21^f^
d > 28	1223.91 ± 139.20	1644.26 ± 230.69	1939.80 ± 313.26	2358.37 ± 463.04
Statistic (F/H)	37.158	16.714	13.022	50.552
*P* value	< 0.001	< 0.001	< 0.001	< 0.001

Data are presented as mean ± standard deviation

Birth weight, weight at birth; Weight at day 28, body weight at postnatal day 28; Weight at 36 weeks PMA, body weight at corrected gestational age of 36 weeks; Discharge weight, body weight at hospital discharge

*P* < 0.05 was considered statistically significant

*TFF* Time to full feeding, *PMA* Postmenstrual age

^a^ *P* < 0.05 compared with the d > 28 group

^b^ *P* < 0.05 compared with the 21 < d ≤ 28 group

^c^ *P* < 0.05 compared with the d > 28 group

^d^ *P* < 0.05 compared with the d > 28 group

^e^ *P* < 0.05 compared with the 21 < d ≤ 28 group

^f^ *P* < 0.05 compared with the d > 28 group

**Table 11:** Comparison of head circumference growth among different TFF groups in very low birth weight infants (1000–1499 g)

TFF (days)	Birth HC (cm)	HC at day 28 (cm)	HC at 36 weeks PMA (cm)	Discharge HC (cm)
7 < d ≤ 14	27.47 ± 1.24^ab^	29.49 ± 1.23	30.52 ± 1.17	31.03 ± 1.09^c^
14 < d ≤ 21	27.07 ± 1.26^a^	29.28 ± 1.37	30.62 ± 1.26	31.21 ± 1.16^c^
21 < d ≤ 28	26.76 ± 1.61	28.86 ± 1.42	30.25 ± 1.03	31.25 ± 1.14
d > 28	26.55 ± 1.96	29.04 ± 1.52	30.91 ± 1.46	31.81 ± 1.53
Statistic (F/H)	42.647	2.039	5.705	17.719
*P* value	< 0.001	0.108	0.127	< 0.001

Data are presented as mean ± standard deviation

Birth HC, head circumference at birth; HC at day 28, head circumference at postnatal day 28; HC at 36 weeks PMA, head circumference at corrected gestational age of 36 weeks; Discharge HC, head circumference at hospital discharge

*P* < 0.05 was considered statistically significant

*TFF* Time to full feeding, *HC* Head circumference, *PMA* Postmenstrual age

^a^ *P* < 0.05 compared with the d > 28 group

^b^ *P* < 0.05 compared with the 21 < d ≤ 28 group

^c^ *P* < 0.05 compared with the d > 28 group

## Discussion

In this multicenter cohort of very and extremely low birth weight infants, we found that delayed achievement of full enteral feeding (FEN) in VLBWI was independently associated with lower gestational age, lower birth weight, and treatment-requiring hsPDA. Moreover, prolonged time to FEN was strongly associated with increased risks of major neonatal morbidities and biochemical evidence of cholestasis. In contrast, no significant determinants or outcome associations were observed in ELBWI. These findings provide additional real-world observational evidence regarding the relationship between feeding progression, physiological immaturity, and short-term neonatal outcomes in VLBWI and ELBWI.

Gestational age and birth weight emerged as the most robust predictors of time to FEN in VLBWI, which is consistent with prior studies reporting that physiological immaturity strongly influences enteral feeding tolerance in preterm infants [17]. Lower gestational age is associated with delayed gastrointestinal motility, impaired intestinal barrier function, and altered immune responses, all of which predispose infants to feeding intolerance and necessitate more cautious advancement of enteral nutrition. Similarly, lower birth weight often reflects a combination of prematurity and intrauterine growth restriction, both of which have been linked to delayed transition from parenteral to enteral nutrition [18]. Our findings reinforce the concept that biological immaturity remains a fundamental constraint on early feeding progression in VLBWI. From a clinical perspective, these findings reinforce the need for stratified feeding strategies based on maturity rather than a one size fits all advancement protocol.

Treatment-requiring hsPDA was independently associated with prolonged TFF in VLBWI. Hemodynamically significant PDA can compromise systemic and mesenteric perfusion through ductal steal, potentially increasing the risk of gut hypoperfusion and feeding intolerance [19]. Consequently, clinicians often adopt more conservative feeding strategies in infants with hsPDA, particularly during periods of cardiorespiratory instability or pharmacological treatment. However, due to the retrospective nature of the study, we could not reliably distinguish whether delayed feeding progression was driven predominantly by the underlying hemodynamic instability or by temporary feeding restrictions during pharmacological treatment. Therefore, hsPDA likely represents a state of hemodynamic instability that delays enteral nutrition rather than acting as an isolated factor. Our findings highlight the importance of optimizing cardiovascular stability and individualized PDA management to facilitate safer progression toward full enteral nutrition.

We further observed that delayed FEN in VLBWI was associated with significantly higher rates of NEC, BPD, late-onset sepsis, and sROP. Several mechanisms may underlie these associations. Prolonged reliance on parenteral nutrition increases exposure to central venous catheters, thereby elevating the risk of bloodstream infections [20]. Ariel A. et al. found that achieving total enteral nutrition at an early stage reduces the incidence of LOS and NEC [21]. This is consistent with our study. Inadequate early nutrient delivery may also adversely affect lung and retinal development, contributing to the pathogenesis of BPD and severe ROP. ^22,23^ However, it is important to acknowledge that delayed TFF may function not only as a contributing factor but also as a surrogate marker of overall illness severity. Infants who are clinically unstable or burdened by multiple comorbidities are more likely to experience delayed feeding advancement and adverse outcomes simultaneously. Therefore, the observed associations should be interpreted as reflecting a complex interplay between nutrition, physiological immaturity, and disease severity rather than a simple causal pathway. Consistent with prolonged parenteral nutrition exposure, VLBWI with delayed FEN exhibited significantly higher peak levels of ALP, ALT, and conjugated bilirubin, suggesting an increased risk of parenteral nutrition–associated cholestasis and hepatic stress. These biochemical alterations underscore the potential metabolic consequences of delayed enteral feeding and further support efforts to minimize the duration of parenteral nutrition whenever clinically feasible.

In contrast to VLBWI, no significant predictors of TFF or associations between TFF and short-term outcomes were identified in ELBWI. Several explanations may account for this finding. First, the relatively smaller sample size of ELBWI in our cohort may have limited statistical power to detect meaningful associations. Second, ELBWI represent a population with profound physiological immaturity and uniformly high baseline risk, in whom feeding advancement is universally cautious and prolonged, resulting in limited variability in TFF. Third, clinical management strategies in ELBWI are often highly standardized and conservative, potentially attenuating the observable impact of individual risk factors. Therefore, the absence of statistically significant associations in ELBWI should not be interpreted as evidence of a true lack of relationship, but rather as a finding that may have been influenced by limited sample size and clinical homogeneity.

Although infants with prolonged TFF exhibited higher discharge weight and head circumference, these findings should not be interpreted as evidence of nutritional benefit associated with delayed feeding advancement. Infants in the prolonged TFF group had substantially longer hospitalization duration, which likely provided greater opportunity for nutritional rehabilitation and catch-up growth before discharge. In addition, growth outcomes were assessed using absolute anthropometric measurements rather than standardized z-scores adjusted for postmenstrual age, which limits the comparability of growth trajectories across groups. Therefore, the observed differences in discharge growth parameters should be interpreted cautiously. Consequently, higher discharge weight and head circumference in infants with prolonged TFF may largely reflect longer inpatient nutritional support rather than improved growth efficiency.

The association between delayed achievement of full enteral nutrition (FEN) and adverse neonatal outcomes is likely complex and bidirectional. In our cohort, delayed FEN in VLBWI was associated with significantly higher rates of NEC, BPD, late-onset sepsis, and sROP, as well as elevated cholestasis-related biochemical markers. Several mechanisms may contribute to these associations. Prolonged dependence on parenteral nutrition and central venous access may increase the risk of bloodstream infection, cholestasis, and metabolic complications. Previous studies have also suggested that earlier establishment of enteral nutrition may reduce the incidence of NEC and late-onset sepsis [20, 21]. Inadequate early nutrient delivery may further impair lung and retinal development, potentially contributing to BPD and severe ROP [22, 23].

However, these associations should not be interpreted as purely causal. Severe clinical instability, including feeding intolerance, cardiorespiratory compromise, systemic inflammation, and hemodynamic disturbances, may itself delay advancement of enteral feeding. Consequently, delayed time to full feeding (TFF) likely reflects overall physiological immaturity and illness severity rather than functioning solely as an independent determinant of adverse outcomes. Therefore, TFF may be better understood as a clinically meaningful marker of neonatal instability and disease burden, while prolonged parenteral nutrition exposure may simultaneously contribute to subsequent infectious and metabolic complications.

Although this study benefited from a multicenter design, a relatively large cohort, and comprehensive evaluation of clinical outcomes, several limitations should be acknowledged. First, the retrospective observational design precludes causal inference and remains susceptible to residual confounding. Delayed achievement of full enteral feeding may reflect underlying illness severity and clinical instability rather than an independent causal determinant of adverse outcomes. Second, feeding practices were not completely standardized across participating centers, and several clinically relevant nutritional variables—including human milk exposure, donor milk use, feeding fortification practices, feeding interruption criteria, feeding advancement rates, and duration of parenteral nutrition—were not consistently available in the retrospective database and therefore could not be incorporated into the analyses. These unmeasured factors may have influenced feeding progression and short-term outcomes. In particular, prolonged parenteral nutrition exposure is an important contributor to cholestasis and hepatic dysfunction in preterm infants; therefore, the observed associations between delayed FEN and elevated cholestasis-related biochemical markers should be interpreted cautiously. Third, growth outcomes were assessed using absolute anthropometric measurements rather than standardized z-scores adjusted for postmenstrual age, which may have limited comparability between groups with different hospitalization durations. In addition, the ELBWI subgroup was relatively small, potentially limiting statistical power to detect significant associations. Therefore, findings from the ELBWI analyses should be interpreted cautiously. Finally, because infants who died before achieving full enteral feeding were excluded, survivor bias may have influenced the observed associations, particularly in the ELBWI subgroup.

In conclusion, our findings indicate that delayed achievement of full enteral nutrition in VLBWI is closely associated with developmental immaturity, hemodynamic instability, and increased short-term morbidity. Time to FEN may serve as a clinically meaningful marker of physiological instability and illness severity rather than an isolated causal determinant of adverse outcomes. These findings provide real-world observational evidence that may help inform individualized nutritional management in preterm infants. Prospective multicenter studies with standardized feeding protocols are warranted to further clarify the relationship between feeding progression and neonatal outcomes.

## Data Availability

The datasets used and/or analyzed during the current study are available from the corresponding author on reasonable request.

## Abbreviations

ALP: Alkaline phosphatase
ALT: Alanine aminotransferase
BPD: Bronchopulmonary dysplasia
CB: Conjugated bilirubin
CGA: Corrected gestational age
ELBWI: Extremely-low-birth-weight infants
FEN: Full enteral feeding
GA: Gestational age
LONS: Late-onset neonatal sepsis
NEC: Necrotizing enterocolitis
NRDS: Neonatal respiratory distress syndrome
PDA/hsPDA: (Hemodynamically significant) patent ductus arteriosus
ROP/sROP: Retinopathy of prematurity/severe ROP
SGA: Small for gestational age
TFF: Time to full enteral feeding
VLBWI: Very-low-birth-weight infants
